# Regulation of Mitophagy by Sirtuin Family Proteins: A Vital Role in Aging and Age-Related Diseases

**DOI:** 10.3389/fnagi.2022.845330

**Published:** 2022-05-09

**Authors:** Wei Wan, Fuzhou Hua, Pu Fang, Chang Li, Fumou Deng, Shoulin Chen, Jun Ying, Xifeng Wang

**Affiliations:** ^1^Department of Anesthesiology, The Second Affiliated Hospital of Nanchang University, Nanchang, China; ^2^Key Laboratory of Anesthesiology of Jiangxi Province, Nanchang, China; ^3^Department of Neurology, The First Affiliated Hospital of Nanchang University, Nanchang, China; ^4^Department of Anesthesiology, The First Affiliated Hospital of Nanchang University, Nanchang, China

**Keywords:** sirtuins, aging, mitophagy, age-related disease, mitochondria, neurodegenerative diseases

## Abstract

Sirtuins are protein factors that can delay aging and alleviate age-related diseases through multiple molecular pathways, mainly by promoting DNA damage repair, delaying telomere shortening, and mediating the longevity effect of caloric restriction. In the last decade, sirtuins have also been suggested to exert mitochondrial quality control by mediating mitophagy, which targets damaged mitochondria and delivers them to lysosomes for degradation. This is especially significant for age-related diseases because dysfunctional mitochondria accumulate in aging organisms. Accordingly, it has been suggested that sirtuins and mitophagy have many common and interactive aspects in the aging process. This article reviews the mechanisms and pathways of sirtuin family-mediated mitophagy and further discusses its role in aging and age-related diseases.

## Introduction

Aging is an inescapable part of human life, and it is a multi-factorial, complex process. During aging, changes in metabolic levels, organ function and gene expression occur in the body, leading to the emergence of several age-related diseases and pathological processes, such as neurodegenerative diseases, cancer, intervertebral disc degeneration (IVDD), and other degenerative changes (Taneike et al., 2010; Rugarli and Langer, 2012; Vara-Perez et al., 2019; Xie et al., 2019). In the past, the treatment of these age-related diseases was mainly focused on treating diseases after they occurred. With the growing awareness of the relevance of the cellular aging process itself to these age-related diseases, there is a growing awareness of the importance of early screening, preventive care, and suppression of specific risk factors (Hou et al., 2019). As the pathways and mechanisms of aging, as well as the greatest risk factors for age-related diseases are being investigated, potential targets for delaying aging and ameliorating age-related diseases have emerged. For example, early restriction of caloric intake in rats prolonged their lifespan and also inhibited the development of age-related diseases (Mccay et al., 1975; Omodei and Fontana, 2011; Madeo et al., 2019). In addition to this, molecular mechanism related to the insulin-like signaling pathway, the target of rapamycin, sirtuins and NAD^+^ has received increasing attention.

As the main ATP-producing organelles of cells, mitochondria play a crucial role in maintaining cellular metabolism and homeostasis. Numerous previous studies have found that mitochondrial dysfunction contributes to the aging process and a variety of age-related diseases (Rugarli and Langer, 2012; Palikaras et al., 2015; Wiley et al., 2016). There are multiple ways to maintain mitochondrial homeostasis and mitochondrial quality control in living organisms, such as mitochondrial biogenesis, fusion, fission and mitophagy, whereby the latter plays an especially important role. Mitophagy is a specialized autophagic pathway that selectively removes damaged and dysfunctional mitochondria, reducing the accumulation of mitochondrial debris and ROS production. Mitophagy is critical for mitochondrial quality control and homeostasis (Ashrafi and Schwarz, 2013). Autophagy is mainly classified into macroautophagy, microautophagy and molecular chaperone-mediated autophagy. Macroautophagy is what we usually call autophagy, which is the main regulatory catabolic mechanism used to degrade long-lived proteins and organelles (Levine and Kroemer, 2008). The process includes the formation and prolongation of the isolation membrane around the cargo, the isolation membrane wrapping around the cargo to form autophagosome, and the fusion of autophagosome with lysosomes to form autolysosome and degrade the cargo (Levine and Kroemer, 2008). mTOR, as a metabolic receptor, is a key and central molecule in the initiation phase of autophagy. mTOR activation negatively regulates the ULK1 complex (a bridge connecting upstream mTOR and downstream autophagosome formation). In addition, ATG genes promote autophagosome formation through ATG12, ATG5 and LC3 complexes (Levine and Kroemer, 2019). Mitophagy, similar to autophagy, involves several processes that recruitment of ubiquitin-autophagy adaptors or activation of mitophagy receptors, recruitment and elongation of isolation membrane/phagophore, autophagosome formation, autophagosome-lysosome fusion, and degradation of autophagosomal contents by lysosomal hydrolases (Tanida, 2011). Different from autophagy, mitophagy can be mediated by various pathways, including ubiquitin-autophagy (PINK/Parkin) and mitophagy receptors (BNIP3, NIX, FUNDC1, etc.) pathways. The specific molecules involved in the mitophagy process will be described following. Interestingly, autophagy and mitophagy sometimes have different or even opposite effects.

The sirtuins (SIRT1-7) are an evolutionarily conserved family of NAD^+^-dependent deacetylases that are involved in a variety of cellular metabolic processes through the deacetylation of target proteins (Table 1) (Chang and Guarente, 2014). The first identified sirtuin protein was the silencing information regulator 2 (SIR2) from *Saccharomyces cerevisiae*. SIR2 was originally described as a chromatin silencing component that represses gene transcription at selected loci (Klar et al., 1979). Growing evidence suggests that sirtuins are not only important energy status sensors, but also protect cells from metabolic stress, regulate the aging process, and alleviate age-related diseases (Chang and Guarente, 2014). Another very important function of sirtuin is to regulate mitophagy. sirtuin members exhibit different cellular localization patterns. SIRT6 and SIRT7 are located in the nucleus and show different subnuclear localization patterns, while SIRT2 is found in the cytoplasm. SIRT1 is located in the nucleus and cytoplasm. Finally, SIRT3, SIRT4, and SIRT5 are localized predominantly in mitochondria (Michishita et al., 2005). Different intracellular localization allows sirtuins to exert different roles. Disruption of mitochondrial membrane potential is a potent trigger of mitophagy, and SIRT3 targets (for instance ATP5O subunit of ATP synthase and complex I) promote flux through oxidative metabolism, generating the electrons needed to restore the proton gradient, playing a role in maintaining mitochondrial membrane potential in response to mitochondrial stress (Ahn et al., 2008; Yang et al., 2016).

**Table 1:** Properties and functions of SIRT famliy related with aging.

**Sirtuins**	**Cellular localization**	**Activity**	**Regulated molecules**	**Related pathologies**	**Functions in aging**
SIRT1	Nucleus and cytoplasm	Deacetylase, ADP-ribosyl-transferase	PINK1/Parkin, UCP2, AKT, PGC-1α, FOXO1/3, mTOR, MFN2, AMPK	XPA, A-T, Cardiac dysfunction, Cardiac remodeling, Neurodegeneration, Ischemia/reperfusion injury, Osteoarthritis, Osteoporosis, Kidney fibrosis etc.	Lifespan, extension, Oxidative stress, DNA repair, Cell cycle arrest
SIRT2	Cytoplasm	Deacetylase, Demyristoylase	PINK1/Parkin, PGC-1α, FOXO3a	Neurodegeneration, Intervertebral disc degeneration	Cell cycle regulation, Longevity, Genome stability
SIRT3	Mitochondria	Deacetylase, Decrotonylase	PINK1/Parkin, FOXO3a, AMPK, PGC-1α, MnSOD	Cardiac aging, Ischemia/reperfusion injury, Diabetes complications, Osteoarthritis, liver injury, Neuronal inflammation	Mitochondrial function, Oxidative stress, longevity
SIRT4	Mitochondria	Deacetylase, ADP-ribosyl-transferase, Lipoamidase	OPA1	Mitochondrial function	Tumor suppression, Apoptosis
SIRT5	Mitochondria	Deacetylase, Desuccinylase, Demalonylase	UCP1	Brown adipose tissue	Oxidative stress
SIRT6	Nucleus (chromatin)	Deacetylase, ADP-ribosyl-transferase, Demyristoylase, Depalmitoylase	AMPK, PGC-1α	Myocardial vulnerability to ischemia/reperfusion injury, Diabetic Cardiomyopathy	Lifespan, extension, Genome stability, Telomere, maintenance
SIRT7	Nucleus (nucleolus)	Deacetylase, Desuccinylase	Histone H3K18	Antagonizes human stem cell aging, Mitochondrial function	Genome stability, Stress resistance

Since sirtuin function and mitochondrial dysfunction both influence the aging phenotype, this review proposes that sirtuin-mediated mitophagy plays a remarkable role in aging and age-related diseases. We further summarize the mechanisms and pathways of mitophagy activation by sirtuins, and explore the relationship between mitophagy, aging and age-related diseases. Finally, this review discusses the problems and development prospects in the research on sirtuin-mediated mitophagy.

## Methods

This review used as keywords SIRT1-7, mitophagy, autophagy, aging and related diseases to select relevant papers listed in the public available PubMed database (https://pubmed.ncbi.nlm.nih.gov/). Exclusion criteria for the references included duplicated research. The PubMed database was accessioned lastly on March 2022.

### The Mechanism of Sirtuin-Mediated Mitophagy

In the ubiquitin pathway, the PINK1/Parkin-dependent ubiquitin pathway is the most extensively studied to date. First, damaged mitochondria need to recruit PINK/Parkin to the mitochondrial surface. In healthy mitochondria, the Ser/Thr kinase PINK1 is maintained at low levels by voltage-dependent proteolysis (Narendra et al., 2010). In mitochondria that sustain damage, mitochondrial membrane potential depolarization effectively triggers mitophagy. PINK1 rapidly accumulates on the surface of mitochondria and forms dimers. Then, the Ser228 and Ser402 residues of PINK1 are autophosphorylated, leading to the recruitment of Parkin to the mitochondrial membrane and activation of its E3 ubiquitin ligase activity (Nguyen et al., 2016a). The ULK1 complex also is recruited to promote the recruitment of Parkin by PINK to the mitochondrial surface by phosphorylating Parkin at Ser108 (Iorio et al., 2021). The next autophagy adapters (p62/SQSTM1, NBR1, NDP52/CALCOCO2, TAX1BP1, and OPTN) play an important role. These autophagy adapters contain both a ubiquitin-binding domain that recognizes ubiquitin chains and an LC3 (a member of the ATG8 family) interaction region (LIR) that acts to recruit phagophore membranes wrapped in LC3 (Zaffagnini and Martens, 2016). Then, phagophore membranes wrapped by the ATG8 family are recruited to the surface of damaged mitochondria by autophagy adapters. Interestingly, OPTN can form directly a complex with ATG9 vesicles (Yamano et al., 2020). NDP52 can also bind directly to the ULK1 complex (Vargas et al., 2019). The cascading reaction: ubiquitylation-OPTN-ATG9/LC3, and ubiquitylation-NDP52-ULK1/LC3. During phagophore membranes elongation to form an isolation membrane, RABGEF1, an upstream factor of the Rab GTPase cascade, is recruited to damaged mitochondria via the ubiquitin chain. RABGEF1 directs Rab proteins Rab5 and Rab7 to damage mitochondria. the Rab cycle assembles ATG9 vesicles and extends the isolation membrane to wrap mitochondria (Heo et al., 2018). In addition, FOXO1 is also thought to increase Rab7 expression. Finally, in autophagosome-lysosome fusion. ATG8 family proteins are associated with elongated isolation membranes and are required for autophagosome-lysosome fusion (Nguyen et al., 2016b).

In the mitophagy receptor pathway, mitophagy receptors on the mitochondrial surface (BNIP3/NIX, FUNDC1, etc.) are activated by stress signals. Similarly, these mitophagy receptors also contain an LIR motif, thereby recruiting the ATG8 family, and mediating the formation of phagophore membranes and isolation membranes. Interestingly, BNIP3/NIX can interact with PINK/Parkin to promote autophagy. NIX is ubiquitinated by Parkin, which in turn promotes the targeting of the mitophagy adapter NBR1 to promote autophagosome formation (Gao et al., 2015). In addition, BNIP3 interacts with PINK1 to promote the accumulation of PINK1 on the outer mitochondrial membrane, leading to the translocation of Parkin to the mitochondria (Zhang et al., 2016).

mTOR, a serine/threonine kinase, is a key factor in the regulation of autophagy and a major regulator of cellular metabolism (Kim and Guan, 2015). The upstream regulator of mTOR is the growth factor/PI3K/AKT signaling pathway. Growth factors such as insulin and IGF activate the PI3K/AKT signaling axis. Activated AKT is directly phosphorylated, thereby activating mTOR. One study suggested that AKT phosphorylation inactivates the downstream effector FOXO transcription factor. And FOXO can inhibit mTOR through multiple mechanisms (Chen et al., 2010). In addition, AMPK, a sensor of cellular energy levels, directly phosphorylates RAPTOR, leading to a decrease in mTOR activity through metastable inhibition (Gwinn et al., 2008). mTOR negatively regulates autophagy by inhibiting the autophagy-initiating ULK complex through phosphorylation of a complex including ATG13 and ULK1/2. In addition, mTOR inhibits ULK1 stability by suppressing the phosphorylation of Beclin-1 regulator 1 (AMBRA1) (Nazio et al., 2013). SIRT1 and SIRT3 can act upstream of the PINK1/Parkin pathway to activate mitophagy, and also via many other pathways. Sirtuins regulate transcription factors and enzymes such as HIF-1α, PGC-1α, FOXO1, PPARγ, etc. Moreover, sirtuins can directly influence mitophagy by interaction with and/or post-translational modification of mitophagy proteins such as ATG5, ATG7, and ATG8. Moreover, they can indirectly increase the expression of mitophagy-related proteins such as mTORC1, PARK1, Beclin-1, BNIP3, etc. (Lee et al., 2008; Huang et al., 2015; Sun et al., 2015). There is growing evidence that sirtuins are key factors in the mitophagy process as well as its effects on aging and age-related diseases.

#### SIRT1 and Mitophagy

**Figure 1:**
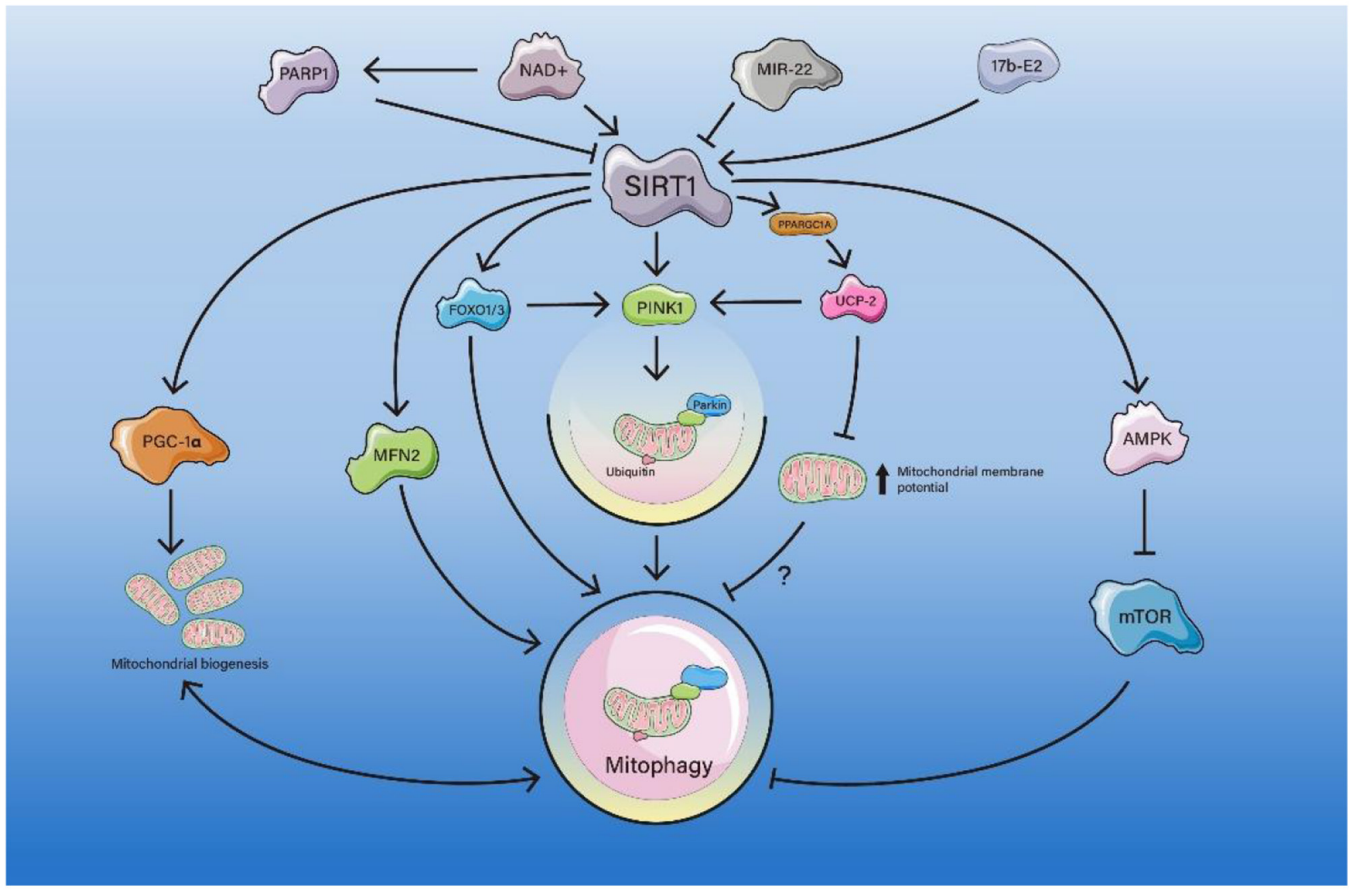
The pathway of SIRT1-mediated mitophagy. PARP1 competes with SIRT1 for NAD+, resulting in inhibition of the SIRT1-PPARGC1A-UCP2 axis, which leads to an increase in mitochondrial membrane potential and inhibition of mitophagy. miR-22 inhibits the SIRT1/PGC-1α axis and decreases PINK1/Parkin expression, suppressing mitophagy. SIRT1 deacetylates FOXO1/3 and enhances mitophagy directly or by activating the FOXO1/3-PINK1-Parkin axis. SIRT1 deacetylates the K655 and K662 sites of MFN2 and enhances mitophagy. 17b-E2 increases SIRT1 and AMPK expression and decrease the expression of mTOR, thus enhancing mitophagy.

In a variety of age-related diseases and pathological processes, such as Alzheimer's disease (AD), cardiac fibrosis and dysfunction, and kidney fibrosis, SIRT1 can active PINK1/Parkin-dependent mitophagy through a variety of pathways (Figure 1), resulting in positive and negative effects on these diseases (Scheibye-Knudsen et al., 2014; Liu et al., 2020b; Wang et al., 2021; Zhao et al., 2021b).

##### PGC-1α Pathway

SIRT1 mediates mitophagy through direct deacetylation of PGC-1α or indirect activation of PGC-1α in a variety of pathological processes.

PGC-1α, as a key factor of mitochondrial biogenesis, can be activated due to deacetylation by SIRT1. PARP1 (a DNA repair enzyme) mediates the loss of SIRT1 activity due to sustained DNA damage response. PARP1 activation depletes NAD+ and attenuates SIRT1 activity. SIRT1 regulates PPARGC1A, and the transcription factors family PPARGC1A/PGC-1α in turn regulates UCP2. UCP2 regulates mitochondrial membrane potential. Therefore, the activation of PARP1 may lead to deactivation of the NAD^+^-SIRT1-PPARGC1A-UCP2 axis and increase the mitochondrial membrane potential, leading to PINK1 cleavage and defective mitophagy (Scheibye-Knudsen et al., 2014). It was also proposed that PARP1 activation can act by attenuating the NAD^+^-SIRT1-PGC-1α axis. Attenuating in SIRT1 regulates the downstream molecule UCP2 through a decrease in deacetylated PGC-1α, leading to depolarization of the mitochondrial membrane potential and an increase in mitochondrial ROS, resulting in defective mitophagy (Fang et al., 2014). PARP1 activation drives an accelerated aging phenotype and this can be partially normalized by pharmacological intervention with PARP1 inhibitors or compounds that increase NAD^+^ (Fang et al., 2014). The PGC-1α pathway can also be activated by SIRT1 in the cardiovascular system. In doxorubicin-induced cardiomyopathy, the role of mitophagy in DOX-induced cardiotoxicity is controversial. Some experiments have shown that DOX inhibits mitophagy and mitochondrial biogenesis (Liu et al., 2019; Wang et al., 2019b, 2021; Xu et al., 2020). Inhibiting miR-22 and knocking out miR-22 affected the SIRT1/PGC-1α pathway and increased expression of PINK1/Parkin to regulate mitochondrial biogenesis and mitophagy, alleviating cardiac fibrosis and cardiac dysfunction (Wang et al., 2021). Specifically, miRNAs repress the expression of their target genes primarily by targeting their 3'untranslated regions (UTR). miR-22 can suppress SIRT1 gene expression through the UTR site, thereby repressing the protein expression levels (Huang et al., 2013). However, there are also reports that DOX can enhance mitophagy by activating the PINK1/Parkin pathway, thus causing cardiotoxic damage (Yin et al., 2018).

Mitophagy elevation might be a protective mechanism against oxidative stress-mediated ROS production through the SIRT1-PGC-1α axis (Liang et al., 2020; Zhao et al., 2022). This axis is also involved in C2C12 myotubes (Chang et al., 2021a), cardiac dysfunction in doxorubicin-induced cardiomyopathy (Wang et al., 2021), and podocyte injury in diabetic nephropathy (Zhou et al., 2019).

##### FOXO1 Pathway

FOXO1 activation by SIRT1 deacetylation can act on multiple processes of mitophagy, such as promoting ULK1 complex formation and isolation membrane elongation.

In cardiac remodeling, some studies have suggested that SIRT1 can deacetylate the transcriptional factor FOXO1 (FOXO proteins play important roles in a variety of intracellular functions including metabolism, stress resistance, longevity and tumor suppression) to activate mitophagy, thereby ameliorating mitochondrial dysfunction and cardiac aging (Ren et al., 2017). FOXO1 increased the expression of Rab7, as a small GTP-binding protein that mediates late autophagosome-lysosome fusion, increasing mitophagy flux (Hariharan et al., 2010). The SIRT 1 activator SRT1720 was found to rescue impaired mitophagy and myocardial contractile function in aging (Ren et al., 2017). In the SIRT1/FOXO1 axis, experiments have suggested that SIRT1-mediated FOXO1 deacetylation and Rab7 upregulation lead to increased starvation-induced autophagy, thus helping to maintain stable cardiac function during starvation (Hariharan et al., 2010).

FOXO1 also interacts with PGC to co-regulate mitochondrial biogenesis and mitophagy. In podocytes from diabetic mice, PGRN (progranulin, a secreted glycoprotein) deficiency exacerbates mitochondrial damage and dysfunction (Zhou et al., 2019). High glucose-induced mitochondrial dysfunction was attenuated by treatment with recombinant human PGRN to enhance mitochondrial biogenesis and mitophagy (Zhou et al., 2019). PGRN maintained mitochondrial homeostasis through PGRN-SIRT1-PGC-1α/FOXO1 axis-mediated mitochondrial biogenesis and mitophagy. SIRT1 increased the DNA-binding ability of FOXO1 by deacetylating its, and potentiated its transcription activity to promote mitophagy via the PINK1/Parkin pathway and thereby protect against podocyte injury under HG conditions (Zhou et al., 2019). In AD, the SIRT1/FOXO axis may also play an important role. The authors of a recent study argued that physical exercise alters the NAD^+^/NADH ratio and enhances expression of SIRT1 in the brain, thereby upregulating mitophagy by activating the FOXO1/3-PINK1-Parkin pathway to attenuate cognitive decline, improve synaptic dysfunction, and decrease the Aβ burden in Alzheimer's disease (Zhao et al., 2021b).

##### AMPK/mTOR Pathway

AMPK/mTOR plays an important role in SIRT1-mediated mitophagy as an energy receptor regulating the activation of the ULK1 complex. Mitophagy also has a non-negligible positive effect on osteoarthritis (OA) and IVDD. Researchers observed reduced mitophagy function of chondrocytes in articular cartilage of patients with osteoarthritis, which accelerated apoptosis and cartilage degeneration (Rockel and Kapoor, 2016). Estrogens, specifically 17b estradiol (17b-E2), treatment for disorders of articular cartilage metabolism and postmenopausal OA. In this model, the RT-PCR results demonstrated that 17b-E2 promotes the expression of SIRT1 mRNA and protein (Mei et al., 2020). 17b-E2 also increases p-AMPK [a metabolic energy sensor, which is activated when the cell energy charge decreases (AMP/ATP ratio increases)], and mitophagy-related proteins, decrease p-mTOR expression, and then activates mitophagy in chondrocytes (Price et al., 2012; Mei et al., 2020). SIRT1 activation can also enhance mitophagy in osteoblasts in osteoporotic rats through the PI3K/AKT/mTOR axis (Yang et al., 2019). In IVDD, the SIRT1-mitophagy axis can also ameliorate intervertebral disc degeneration and high-magnitude compression-induced senescence of nucleus pulposus cells (Xie et al., 2019; Wang et al., 2020b).

Treatment with D-galactose leads to significant senescence of cardiomyocytes, shortened telomeres, increased cellular senescence marker proteins p21 and p53, as well as reduced mitophagy mediated by reduced expression of SIRT1 and PINK1/Parkin in aging mice (Hong et al., 2021). Acacetin enhances the mitophagy proteins PINK1, Parkin, and LC3II via SIRT1-mediated activation of SIRT6 and pAMPK, thereby enhancing mitophagy, reversing the aging-related mitochondrial membrane potential depolarization, and alleviating D-galactose-induced cardiac senescence (Hong et al., 2021).

##### Other Pathways

In ischemia/reperfusion (I/R) injury of the liver, biochemical analysis revealed that the vast majority of losses of both SIRT1 and mitofusin 2 (MFN2) (a mitochondrial outer membrane protein that has diverse functions such as mitochondrial fusion and metabolic regulation) after I/R occurs in old hepatocytes rather than young cells. Co-overexpression of both proteins resulted in SIRT1 deacetylated K655 and K662 residues near the C-terminus of MFN2, leading to the activation of autophagy/mitophagy, which prevented mitochondrial dysfunction and reduced cell death after reperfusion (Biel et al., 2016; Chun et al., 2018). However, the mechanism of how MFN2 enhances mitophagy is not well understood, possibly because MFN2 may be involved in autophagosomes-lysosomes fusion through interaction with the Ras-associated protein Rab7 (Zhao et al., 2012). On the other hand, MFN2 may mediate the recruitment of Parkin to damaged mitochondria. Parkin binds to MFN2 in a PINK1-dependent manner; PINK1 phosphorylates MFN2 and promotes its Parkin-mediated ubiquitination. Excision of MFN2 in mouse cardiomyocytes prevents depolarization-induced Parkin translocation to mitochondria and inhibits mitophagy (Chen and Dorn, 2013). Another study of I/R injury in the liver also proposed that enhancing SIRT1-mediated autophagy can protect against I/R injury (Cho et al., 2017).

In a recent study, cardiomyocytes subjected to H/R were damaged due to excessive reactive oxygen species and decreased mitophagy (Chang et al., 2021b). TMBIM6, a calcium channel-like protein, can interact with calcium signaling proteins to inhibit apoptosis of the endoplasmic reticulum pathway and regulate cell survival and death. Quercetin increased the expression of TMBIM6, while short interfering RNA transfection of SIRT1 further inhibited the expression of TMBIM6. Therefore, treatment with Quercetin regulated mitophagy through the SIRT1/TMBIM6 axis and inhibited H/R-induced oxidative stress-induced damage (Chang et al., 2021b). However, the mechanism of interaction between SIRT1 and TMBIM6 needs to be further elucidated.

In acute kidney injury and kidney fibrosis, enhanced mitophagy can also alleviate related symptoms via the activation of the SIRT1/PINK1/Parkin axis (Gao et al., 2020; Liu et al., 2020b). Important roles of SIRT1-mitophagy have also been found in many other diseases, such as polycystic ovary syndrome (PCOS) (Yi et al., 2020), glioblastoma (Yao et al., 2018), spina bifida aperta (Zhao et al., 2021a), age-related hearing loss (Xiong et al., 2019), and prostatic intraepithelial neoplasia (Di Sante et al., 2015). An increasing number of SIRT1-mediated mitophagy pathways have been identified in various diseases and aging processes, providing a basis and guidance for future therapeutic targets.

#### SIRT3 and Mitophagy

**Figure 2:**
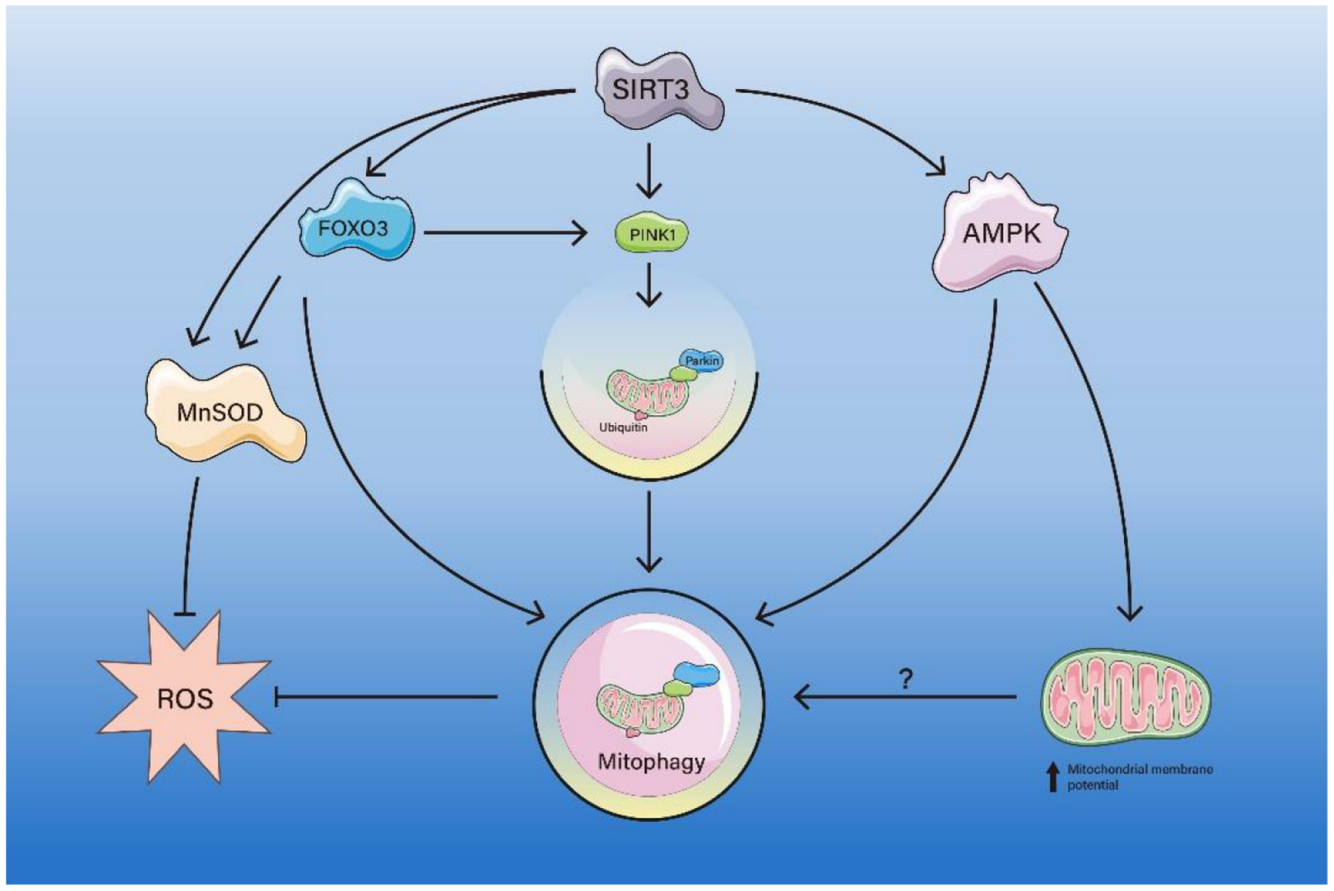
The pathway of SIRT3-mediated mitophagy. SIRT3 deacetylates FOXO3, which activates MnSOD, inhibits ROS production. FOXO3 deacetylated by SIRT3 also enhances mitophagy directly or via the PINK1/Parkin axis, thereby inhibiting ROS production. SIRT3 enhances AMPK activity, thereby directly enhancing mitophagy or indirectly enhancing mitophagy by increasing mitochondrial membrane potential.

SIRT3, similar to SIRT1, acts as an important factor in the resistance to aging of multiple organs and related diseases, and also relies on multiple pathways to activate mitophagy (Figure 2).

##### FOXO3 Pathway

SIRT3, similar to SIRT1, deacetylates FOXO3, thereby activating the ubiquitination-dependent mitophagy pathway. SIRT3-mediated FOXO3a activation also enhances mitochondrial fission and mitophagy through PINK1/Parkin activation and creates a cardioprotective environment during aging (Das et al., 2014). Similarly, there is also a study that suggests that activation of the SIRT3/FOXO3a/Parkin signaling pathway can protect against atherosclerosis (AS) (Ma et al., 2018). Furthermore, a study proposed that SIRT3 activates Parkin-mediated mitophagy through FOXO3a deacetylation, which is beneficial to the regulation of diabetic cardiomyopathy (Yu et al., 2017). Conversely, a study concluded that SIRT3/FOXO3a-mediated excessive mitophagy and autophagy will aggravate anoxia/reoxygenation injury (Wu et al., 2020). In addition, H/R-induced cardiomyocyte injury will activate excessive autophagy, and inhibition of excessive autophagy can significantly reduce H/R damage, thereby improving the survival of cardiomyocytes (Shi et al., 2017). It may be inferred that autophagy and mitophagy have different or even opposite roles in cardiac I/R injury. SIRT3-mediated mitophagy plays a role not only in primary cardiovascular disease, but also in secondary cardiac diseases, such as diabetic cardiomyopathy (DCM). SIRT3 activation upregulates Parkin expression and mitochondrial recruitment, thereby enhancing mitophagy and alleviating the phenotype of DCM (Wang et al., 2019c).

In complications of diabetes, such as diabetic keratopathy (Hu et al., 2019) and diabetic nephropathy (Feng et al., 2018), SIRT3 overexpression promotes wound healing under high glucose (HG) conditions by activating FOXO3a/PINK1/Parkin axis-mediated mitophagy (Hu et al., 2019). Moreover, SIRT3/FOXO3a/Parkin axis-mediated mitophagy activation protects against oxidative liver injury (Chen et al., 2020), neuroprotective (Zhang et al., 2020b), aging (Tseng et al., 2013) and inclusion body myositis (Koo et al., 2019).

##### Reduce ROS

SIRT3 maintains ROS levels in the normal range to protect organisms from oxidative stress-induced pathology (Pi et al., 2015; Katwal et al., 2018). As a mitochondrial fidelity protein, SIRT3 directs energy generation and regulates ROS scavenging proteins by activating direct manganese superoxide dismutase (MnSOD) enzymatic dismutase activity (Tao et al., 2014). SOD2 is acetylated at lysine 68 and 122 and this acetylation decreases SOD2 activity. SIRT3 deacetylates MnSOD, leading to an increase in SOD2 enzyme activity (Tao et al., 2014). In hypertensive cardiac remodeling, SIRT3 deacetylates FOXO3 to activate FOXO3-dependent antioxidants, MnSOD and catalase, while suppressing reactive oxygen species (ROS), thereby blocking the cardiac hypertrophic response. It was found that SIRT3 could promote angiogenesis by enhancing PINK1/Parkin pathway-mediated mitophagy to attenuate mitochondrial dysfunction (Wei et al., 2017).

Immunoprecipitation and western blot assays also suggest that the activity, expression, and deacetylation of mitochondrial MnSOD and PGC-1α were reduced in the aging heart (Li et al., 2018b). It was also found that the expression level of SIRT3 was significantly lower in the myocardium of aged mice compared with that of young mice (Li et al., 2018b). The reduced PGC-1α and MnSOD expression, deacetylation, and activity were improved by SIRT3 (Li et al., 2018b). In addition, the reduction of SIRT3 in the aging myocardium allows for increased acetylation of P53 and binding to Parkin. Acetylated P53 binds to Parkin and blocks its ectopic position, leading to a decrease in mitophagy (Li et al., 2018b). SIRT3 regulates mitochondrial biogenesis and mitophagy while also promoting mitochondrial oxidative stress resistance by altering the acetylation of MnSOD and enhancing its ability to scavenge ROS, thereby attenuating cardiac dysfunction of the aged heart (Zhao et al., 2018).

##### Other Pathways

There are also studies showing that SIRT3 can activate Bnip3 expression and mitophagy through the ERK-CREB signaling pathway to ameliorate non-alcoholic fatty liver disease (Li et al., 2018a). The ERK-CREB signaling pathway is the upstream mediator of mitophagy activation. SIRT3 overexpression increased p-ERK content, p-CREB, and NIBP3 levels, and blockade of the ERK pathway significantly inhibited Bnip3 expression. These data suggest that SIRT3 controls Bnip3 expression through the ERK-CREB signaling pathway (Li et al., 2018a).

The role of SIRT3-mediated mitophagy in cardiac I/R injury is controversial. A recent study suggests that augmenting mitochondrial fusion and activating the AMPK/SIRT3 signaling pathway can increase the mitochondrial membrane potential and improve mitophagy, thereby protecting against cardio-cerebrovascular I/R injury (Liu et al., 2020a). However, the article did not explain the specific mechanism of interaction between AMPK and SIRT3. On the contrary, some studies suggest that in SIRT3+ cells, phosphorylation of LKB1 (an upstream kinase of AMPK) is increased, which increases AMPK activity. Thus, SIRT3 may be enhancing autophagy levels through the LKB1-AMPK-mTOR axis (Zhang et al., 2018; Han et al., 2020). Additional sirtuin family-mediated mitophagy pathways may be discovered in the future, which will provide new ideas for future interventions in related diseases and aging.

#### Other Sirtuins and Mitophagy

As members of the same family, sirtuins share several similar functions, such as deacetylation, and are collectively involved in aging and the progression of related diseases. SIRT2 is the most abundantly expressed sirtuin in the brain, and was found to be expressed exclusively in growth cones of postmitotic cells and cytoplasmatic neuritis (Harting and Knoll, 2010). SIRT2 was considered to act as a tubulin deacetylase that regulates microtubule network acetylation, and overactivation of SIRT2 may result in loss of mitochondrial potential, further leading to a dysfunction in autophagy/mitophagy (Silva et al., 2017). Treatment with the specific SIRT2 inhibitor AK1 treatment or SIRT2 knockout in mice can recover microtubule stabilization and improve autophagy/mitophagy, favoring cell survival in AD by eliminating toxic Aβ oligomers (Silva et al., 2017). SIRT2 can also deacetylate PGC-1α and FOXO3a. In response to oxidative stress, SIRT2 can deacetylate FOXO3a to transcriptionally activate the SOD2 gene and Bim gene, which reduces cellular ROS levels. Furthermore, as Bim is a pro-apoptotic factor, SIRT2 promotes cell death when cells are under severe stress (Wang et al., 2007). Consistent with previous findings, SIRT2-deficient mice exhibited increased oxidative stress, decreased ATP levels and altered mitophagy in brain cells. Mitophagy factor ATG5 may be a SIRT2 downstream deacetylation target. Because a difference in ATG5 acetylation was observed in lysates from the cortex from Sirt2-/- mice (Liu et al., 2017).

SIRT4 is localized to mitochondria. Interestingly, SIRT4 expression may promote stress-induced autophagic flux, but also decrease PINK1/Parkin-associated mitophagy, leading to an increase of mitochondrial content (Lang et al., 2018). SIRT4 activation shifts mitochondrial fusion, resulting in the downregulation of mitophagy during aging via the GTPase OPA1 (L-OPA1), which promotes mitochondrial fusion (Lang et al., 2018).

SIRT5 is considered to be a mitochondrial desuccinylase and demalonylase. In brown adipose tissue, SIRT5-KO elevates protein succinylation of two lysine residues in UCP1, K56 and K151, while reduced function of UCP1 may result in impaired mitochondrial respiration and defective mitophagy (Wang et al., 2019a). However, excess succinylation of UCP1 due to SIRT5 inhibition may induce autophagy/mitophagy and mitochondrial dysfunction (Zhang et al., 2020a). SIRT5 inhibition can also result in glutaminase succinylation to regulate ammonia production. Ammonia can also activate mitophagy, as evidenced by the measurement of mitophagy markers BNIP3 and PINK1/Parkin (Polletta et al., 2015).

A recent study found a role for SIRT6 in diabetic cardiomyopathy (DCM) (Yu et al., 2021). Long-term diabetes reduces cardiac melatonin membrane receptor expression and decreases myocardial SIRT6 and AMPK-PGC-1α-AKT signaling. AMPK appears to alleviate DCM and improve mitochondrial quality control through multiple pathways. Activation of the AMPK-PGC-1α pathway reduced mitochondrial division in the diabetic heart by inhibiting Drp-1 phosphorylation. AMPK-PGC-1α-mediated activation of Nrf1-Tfam (promoter of the nuclear gene encoding a subunit of the mitochondrial oxygen-phosphate complex) promoted mitochondrial biogenesis in the diabetic heart. AMPK also enhanced mitophagy through the ULK1 pathway (Yu et al., 2021). Melatonin treatment enhances mitophagy and mitochondrial biogenesis by activating SIRT6 and AMPK-PGC-1α-AKT signaling, thereby inhibiting the progression of DCM and subsequent myocardial ischemia-reperfusion injury (Yu et al., 2021).

Although there is no evidence of direct induction of mitophagy by SIRT7, a study has shown that SIRT7 can act as a histone H3K18-specific deacetylase to indirectly affect mitophagy by controlling mitochondrial biogenesis, ribosomal biosynthesis, and DNA repair (Yan et al., 2018). Furthermore, deficiency of SIRT7 results in a loss of heterochromatin and accelerates human mesenchymal stem cell (hMSC) senescence (Bi et al., 2020). Overall, the mechanisms through which other sirtuins mediate mitophagy are not well understood, but remain highly relevant to mitochondrial function. Accordingly, there are many possible mechanisms waiting to be studied in the future.

### Mitophagy in Aging and Age-Related Diseases

**Figure 3:**
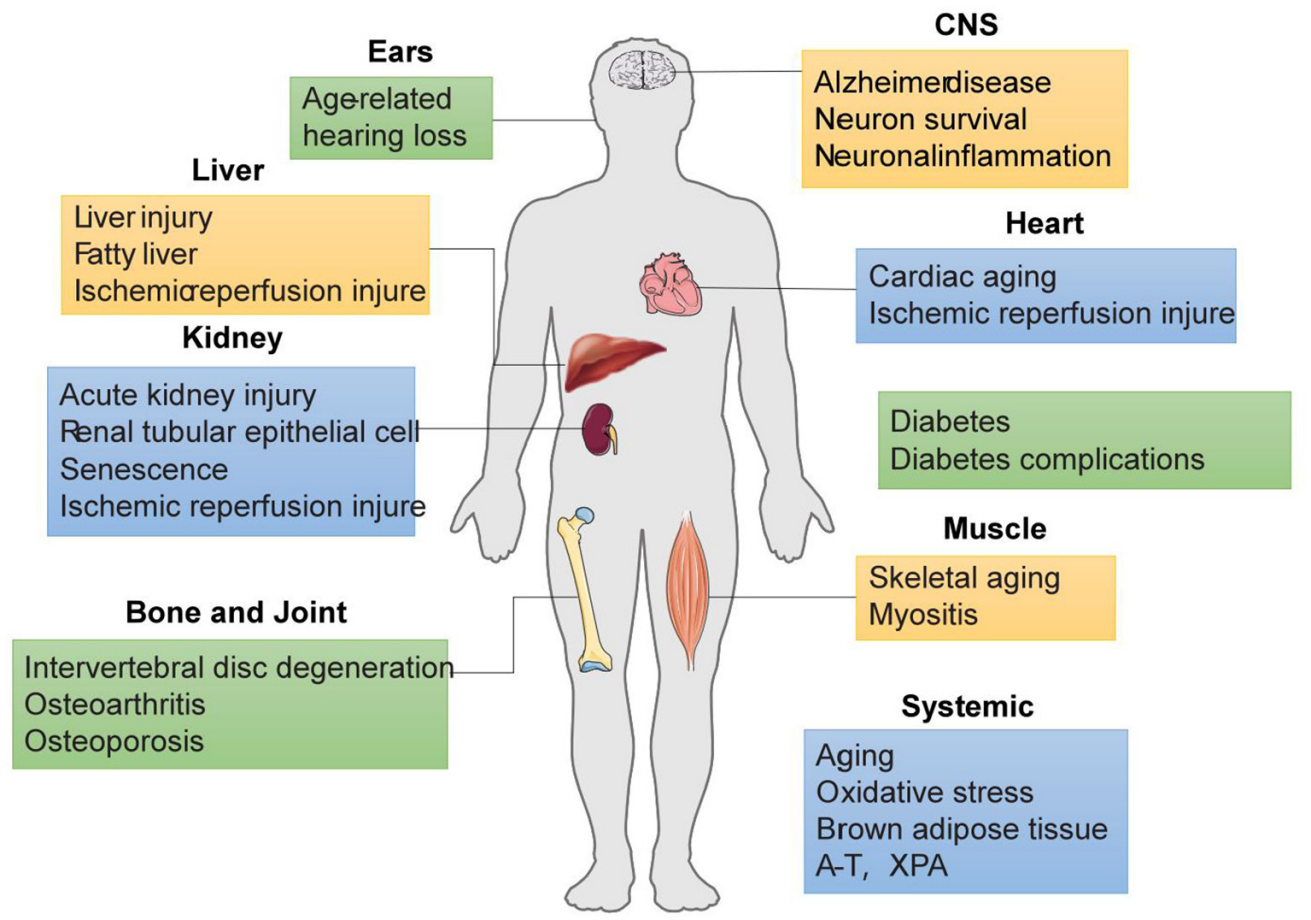
SIRT-medidated mitophagy in age-related pathologies.

Healthy mitochondria are essential for many basic cellular processes, including energy production, metabolite synthesis, and lipid metabolism. Mitochondrial mass is mainly regulated by mitophagy. When mitophagy is impaired, mitochondrial dysfunction leads to impaired energy homeostasis and ultimately cellular dysfunction, with implications for aging and multiple age-related diseases, such as cancer, cardiovascular diseases, neurodegenerative diseases and senile osteoporosis (Figure 3) (Palikaras et al., 2015; Fang et al., 2016; Zhang et al., 2017; Ziegler et al., 2018; Fang, 2019; Chen et al., 2021; Guo et al., 2021).

#### Mitophagy Dysfunction Leads to Cellular Senescence

In a previous study, it was found that autophagy can extend the life span of *Saccharomyces cerevisiae* (Alvers et al., 2009). Subsequently, autophagy was also found to be required for lifespan extension in Caenorhabditis elegans and *Drosophila melanogaster* with reduced food intake, TOR or insulin/IGF-1 signaling (Hars et al., 2007; Simonsen et al., 2008; Toth et al., 2008; Alvers et al., 2009). Knockdown of the Atg7 and Atg12 genes or depletion f Beclin1 suppressed the long-lived phenotype of wild-type and longevity gene mutant nematodes (Toth et al., 2008). This study implies that autophagy is the common downstream process of distinct longevity-related pathways. Furthermore, specific enhancement of ATG8 expression in brain extends the average lifespan of fruit flies and promotes resistance to oxidative stress (Simonsen et al., 2008). This result implies that the effects of autophagy on aging at different sites are different. Although a large body of past research suggests that autophagy inhibits strongly aging, it does not reveal the pathways through which autophagy affects aging and the specific roles played by various types of autophagy.

Subsequent studies found that deregulation of cellular mitochondrial content is the common denominator of aging and numerous pathological conditions (Artal-Sanz and Tavernarakis, 2009; Kaeberlein, 2010). Mitochondria gradually accumulate with age in wild-type *C. elegans*, and depletion of autophagy regulators may be the main reason for this phenomenon (Palikaras et al., 2015). This led to further studies focusing on the contribution of mitophagy to longevity. When DCT-1 (a putative ortholog to the mammalian NIX/BNIP3) and PINK1 are knocked down in wild-type strains and long-lived mutants, the lifespan extension of mutants with moderate mitochondrial dysfunction or that caused by caloric-restriction is abrogated. Furthermore, DCT-1 and PINK-1 mutants are substantially more sensitive to various stressors (Palikaras et al., 2015). Mitophagy-deficient animals exhibit decreased ATP levels, increased mitochondrial reactive oxygen species (mROS) production, mitochondrial membrane depolarization, and increased oxygen consumption, which are exacerbated under stress (Palikaras et al., 2015).

Cellular senescence is a cell state triggered by stressful insults and certain physiological processes, characterized by a prolonged and generally irreversible cell-cycle arrest with secretory features, macromolecular damage, and altered metabolism (Gorgoulis et al., 2019). Senescent cells exhibit a powerful secretory activity, known as the senescence-associated secretory phenotype (SASP), which can lead to a positive feedback to accelerate cellular senescence and affect the local microenvironment of cells, thereby possibly influencing the progression of aging and related diseases (Zlotorynski, 2020). Although there are some differences in SASP between different tissues and aging models, the core components of SASP are mainly pro-inflammatory interleukin-6 (IL-6), CXC chemokine ligand 8 (CXCL8) and monocyte chemoattractant protein 1 (MCP1) (Coppe et al., 2008). Mitochondrial dysfunction will induce cellular senescence and result in features of aging, including arrested growth and SASP. There is evidence that mitochondrial dysfunction-induced SASP differs from many other aging models, due to a lack of SASP-specific mRNAs encoding IL1B, IL-6 and IL-8, along with high levels of mRNAs encoding the other SASP factors, such as AREG and IL-10 (Wiley et al., 2016). This indicates that mitochondrial dysfunction causes a senescent phenotype that differs from that caused by other senescence inducers. Similarly, widespread targeted depletion of mitochondria through mitophagy blocks the expression of features typical of cellular senescence, such as the pro-inflammatory and pro-oxidant SASP and changes in the expression of the cyclin-dependent kinase inhibitors p16 and p21, while still preserving the cell cycle arrest (Correia-Melo et al., 2016).

However, the mechanism through which mitochondrial dysfunction leads to senescence is controversial. Defective mitophagy can lead to the accumulation of dysfunctional mitochondria and mROS, which in turn can damage nuclear DNA, thereby activating a DNA damage response that induces senescence (Moiseeva et al., 2009; Passos et al., 2010). However, some experiments indicate that antioxidants which reduce mitochondrial ROS in mitochondrial dysfunction-induced senescent cells do not prevent the growth arrest or focal enhancement of the DNA damage marker 53BP1 (Wiley et al., 2016). This study suggests that a decrease of the NAD^+^/NADH ratio due to mitochondrial dysfunction activates AMPK and p53, thereby inducing cellular senescence (Wiley et al., 2016). Dysfunctional mitochondria can also promote senescence and SASP through the mitochondria-to-nucleus retrograde signaling pathway. The increase of cytoplasmic chromatin fragments (CCFs), which are extruded from the nucleus and recognized by the innate immunity cytosolic-DNA sensing cGAS–STING pathway in senescent cells, will activate the SASP and pro-inflammatory genes. Specifically, the cytosolic DNA sensor CGA generates a second messenger loop GMP-AMP (cGAMP) that binds and activates the bridging protein STING, which recognizes CCFs (Vizioli et al., 2020). The cGAS–STING pathway leads to activation of NF-κB signaling that turns on transcription of proinflammatory genes (Vizioli et al., 2020). Although the clearance of mitochondria through mitophagy in senescent cells did not revert growth arrest, it significantly suppressed CCFs and the SASP (Vizioli et al., 2020). This suggests that mitochondrial dysfunction can promote the SASP and inflammation by activating a signaling pathway that transduces to the nucleus. Aging is a complex process involving multiple triggers, and there may be multiple pathways that work together, which will need to be resolved in the future.

#### Regulation of Mitophagy During Aging by Sirtuins

The role and function of the sirtuin family in aging has much in common with the function of mitophagy in aging, and sirtuins can positively or negatively regulate mitophagy through multiple pathways. Therefore, it is conceivable that there are many intersections between these processes and that they influence each other. For instance, sirtuins induce and maintain mitophagy, and thus regulate mitochondrial homeostasis to mitigate the aging phenotype.

In a study of cardiac aging, the aging process was found to disrupt mitophagy and mitochondrial integrity, as evidenced by decreased levels of beclin-1, Atg7, LC3B, BNIP3, PINK1, Parkin, UCP-2, and PGC-1α activity, increased phosphorylation of AKT and the nuclear transcriptional factor FOXO1, as well as the increased acetylation of FOXO1 (Ren et al., 2017). Importantly, Akt2 ablation prolonged the lifespan and alleviated aging-induced unfavorable changes of myocardial function. Rapamycin and the SIRT1 activator SRT1720 improved aging-induced contractile dysfunction and mitophagy of cardiomyocytes, the effects of which were reversed by Akt2 activation (Ren et al., 2017). This indicates that Akt2 ablation protects against cardiac aging by restoring SIRT1 and FOXO1-related mitophagy and mitochondrial integrity. However, recent experiment suggests that aging upregulates sirtuin levels in skeletal and cardiac muscle, but enhances protein acetylation (Yeo et al., 2020). These inconsistencies may reflect differences between species, organs, and methods used. As aging activates PARP1 and CD38, both enzymes compete with sirtuin for NAD^+^, resulting in aging muscles showing significant signs of mitophagy dysfunction, mitochondrial dysfunction and oxidative stress (Yeo et al., 2020).

Another experiment found significantly lower levels of SIRT3 expression in the myocardium of aged mice. The myocardium of SIRT3 knockout mice exhibited significant aging characteristics, including mitochondrial protein dysfunction, enhanced oxidative stress and energy metabolism dysfunction (Li et al., 2018b). The levels of senescence marker genes p16 and p53 were upregulated by about 80 and 140%, while both the β-galactosidase^+^ cell ratio and lipofuscin content were increased by about 50%. SIRT3 deficiency increased the level of P53 acetylation and affected Parkin-mediated mitophagy through increased p53-Parkin binding. Importantly, therapeutic activation of SIRT3 and improvement of mitochondrial function may alleviate the symptoms of cardiac aging (Li et al., 2018b).

Resveratrol, a polyphenolic antioxidant and sirtuin activator, exerts a cardioprotective anti-aging effect through the activation of SIRT1/3-Parkin-mediated mitophagy (Das et al., 2014). Resveratrol also reduces aging in other tissues. In adipose tissue, targeted activation of SIRT3 by epigallocatechin gallate (EGCG) and resveratrol significantly reduced IL-6 secretion, regulated ROS through different pathways, and ultimately delayed cellular senescence and senescence-induced inflammatory processes (Lilja et al., 2020). Similarly, advanced glycation end products (AGEs) significantly aggravated the senescence of bone marrow mesenchymal stem cells (BMSCs), while also inhibiting mitophagy and promoting mitochondrial dysfunction. Importantly, SIRT3 silencing may further strengthen this effect (Guo et al., 2021). Subsequently, overexpression of SIRT3 by intravenous injection of a recombinant adeno-associated virus 9 carrying SIRT3 plasmids to improve mitophagy significantly alleviated BMSC senescence (Guo et al., 2021). Sirtuin-mediated mitophagy also plays an important role in the aging of many other organs and tissues, such as I/R injury in aged livers (Chun et al., 2018), renal tubular epithelial cell senescence (Liu et al., 2020b) and aging-associated neuronal inflammation (Huang et al., 2019). Targeted therapeutic options are proposed for these aging and age-related diseases, such as the activation of sirtuins or the targeting of upstream and downstream molecules. While specific clinical effectiveness remains to be studied, this research area offers exciting new directions for extended life span.

#### Mitophagy in Neurodegenerative Diseases

It is well known that neurodegenerative diseases are closely related to aging and that dysfunctional mitochondria also accumulate in aging and neurodegenerative diseases. This implies that mitochondria play an important role in maintaining neuronal functional homeostasis and the progression of age-related neurodegenerative diseases. Mitochondrial dysfunction due to impaired mitophagy leads to ROS accumulation and elevated cytoplasmic calcium levels, which may trigger apoptotic and necrotic cell death cascades, leading to cellular stress and eventually to neurodegeneration (Rugarli and Langer, 2012; Huang et al., 2022). To date, multiple studies have linked mitochondrial dysfunction to age-related neurodegenerative diseases, such as Alzheimer's, Parkinson's and Huntington's disease.

Alzheimer's disease (AD) is one of the most common neurodegenerative diseases, and it is characterized by cognitive dysfunction and memory loss as the main symptoms. Currently recognized pathological hallmarks include extracellular deposits of Aβ and intraneuronal accumulation of hyperphosphorylated Tau protein, which can promote mitochondrial defects. Conversely, mitochondrial dysfunction may contribute to the pathologies related to Aβ and hyperphosphorylated Tau (p-tau) (Kerr et al., 2017). In an APP/PS1 mouse model, this vicious cycle can be broken by activating mitophagy. Mitophagy activation can not only diminish Aβ1–42 and Aβ1–40 and prevent cognitive impairment through microglial phagocytosis of extracellular Aβ plaques, but also abolishes Tau hyperphosphorylation in human neuronal cells and prevents memory impairment in transgenic tau nematodes and mice (Fang et al., 2019). However, it is still unclear if mitophagy is a cause or a consequence of Aβ and Tau, with questions remaining as to the chronological order of their occurrence in AD models. Interestingly, physical exercise significantly alters the NAD^+^/NADH ratio, activating mitophagy through the SIRT1-PINK1/Parkin pathway, thereby attenuating mitochondrial dysfunction and Aβ-induced cognitive decline in AD animal models (Zhao et al., 2021b). While it cannot be excluded that physical exercise may act on AD through pathways other than the mitophagy pathway, these results suggest a new mechanism through which exercise can be good for the body.

Other member of the sirtuin family, such as SIRT2, also exacerbates AD through attenuating mitophagy which leads to the accumulation of Aβ and dysfunctional mitochondria. In cells containing mtDNA from AD patients, it was found that elevated SIRT2 levels, loss of mitochondrial membrane potential and impaired mitophagy processes. SIRT2 loss of function recovers microtubule stabilization and improves mitophagy, thereby eliminating toxic Aβ oligomers and increasing cell survival (Silva et al., 2017). Importantly, a recent study found that mitochondrial debris released from microglia can trigger the A1 astrocytic response, resulting in the propagation of the inflammatory response and neuronal cell death, linking dysfunctional mitochondria and glial cells in the brain and suggesting a potential new intervention for neurodegeneration (Joshi et al., 2019). Interestingly, a recent ad study showed that Honokiol (HKL, an extract from bark of Magnolia) can improve the activity of SIRT3 and improve the synaptic damage, mitophagy and mitochondrial dysfunction of hippocampal neurons in a SIRT3 dependent manner, thus exerting anti-AD effect (Hou et al., 2022).

Factors in the development of Parkinson's disease (PD) include mainly genetic and environmental factors, and aging is one of the major risk factors. Genetic risk is usually divided into 1) 5% of cases are familial Parkinson's disease, carrying heritable, disease-related single gene mutations (such as Parkin, PINK and α-synuclein) and 2) 95% of cases are sporadic Parkinson's disease, with more common but less effective genetic variants, often acting as one of the risk factors in combination with multiple factors such as the environment (Subramaniam and Chesselet, 2013; Barazzuol et al., 2020; Blauwendraat et al., 2020). Previous studies have found that familial Parkinson's disease may be associated with mutations in the PINK1 and Parkin genes (Subramaniam and Chesselet, 2013). The PINK1/Parkin axis plays a significant role in the removal of dysfunctional mitochondria, which implies that mitophagy plays a non-negligible role in PD. Some studies suggest that USP30, a deubiquitinase localized to mitochondria, inhibits mitophagy mediated by protein kinase PINK1 and the ubiquitin ligase Parkin. Thus, USP30 deficiency activates PINK1/Parkin-mediated mitophagy to protect against motor disabilities, ameliorate defects in dopamine levels and enhance survival upon oxidative stress in PD models (Bingol et al., 2014). Mitochondrial dysfunction was also found to be associated with neuronal inflammation, while mitochondria-induced neuronal death has been reported as evidence of ongoing neurodegenerative disease (Huang et al., 2019). Parkin-mediated mitophagy can be activated by mitochondria acid 5 (MA-5) to attenuate neuroinflammation by reducing mitochondrial damage and promoting cell survival (Huang et al., 2019).

Huntington's disease (HD) is also a neurodegenerative disease with similar features, such as progressive neuronal loss and the presence of pathogenic forms of misfolded protein aggregates (mutant huntingtin mHTT) (Sonsky et al., 2021). The HD model is characterized by a large number of dysfunctional mitochondria. Altered expression of Sirtuins is found in HD models and affects mitochondrial dynamics (including mitophagy) (Neo and Tang, 2018; Franco-Iborra et al., 2021; Naia et al., 2021; Sonsky et al., 2021). Overexpression of SIRT1 and SIRT3 promotes antioxidant effects in HD cells and deacetylates PGC-1α and FOXO3, thereby enhancing mitochondrial function, biogenesis, and mitophagy (Manjula et al., 2020; Naia et al., 2021). Moreover, mHTT can inhibit multiple steps of the process of mitophagy, such as ULK1 activation, mitophagy receptor recruitment, and LC3-mitophagy receptor interactions (Franco-Iborra et al., 2021).

Mitophagy also seems to play a role in other less prevalent neurodegenerative diseases, such as amyotrophic lateral sclerosis (ALS). Mitophagy adapter OPTN is phosphorylated by TBK1, which enhances the binding affinity between ubiquitin chain and Atg8 family proteins and promotes the recruitment of isolation membrane to mitochondria. The loss of function of OPTN and TBK1 (ALS gene mutation site) will lead to the impairment of mitochondrial phagocytosis and the accumulation of damaged mitochondria (Evans and Holzbaur, 2019). Although the specific mechanism is not clear, it is interesting to be a potential therapeutic target for ALS.

## Conclusions and Future Perspectives

Since sirtuins were found to extend the lifespan of *Saccharomyces cerevisiae* and *Caenorhabditis elegans*, the mechanism of sirtuin lifespan extension and whether it can extend the lifespan of other species has been actively studied. With increasing research in the last 5 years, sirtuins are increasingly recognized as being critical for regulating mitophagy and maintaining mitochondrial homeostasis. Taken together, the sirtuin family can activate or inhibit mitophagy through multiple pathways, for instance deacetylation of PGC-1α and FOXO1/3 and reduction of ROS, thereby affecting aging and age-related diseases. By targeting these pathways, it may be possible to delay aging.

A consensus has now emerged from many studies of sirtuin activators that sirtuins mediated aspects of caloric restriction (Canto and Auwerx, 2009). Sirtuin activators can modulate aging and age-related diseases by activating a variety of sirtuin-induced biological functions, and have demonstrated significant aging delay and disease mitigation in experimental models (Table 2). Excitingly, some sirtuin activators are already in clinical trials. For example, resveratrol acts in neurological diseases (NCT02621554, NCT02336633, NCT00678431), SRT2104 in inflammation (NCT01453491, NCT01154101), and nicotinamide riboside in the cardiovascular system (NCT02678611). Furthermore, decreased NAD^+^ levels during aging reduce sirtuin activity, which may contribute to the aging process.

**Table 2:** The modifiers of sirtuins.

**Sirtuin activators**	**Sirtuin effect**	**Experimental setting**	**References**
NAD+	Activates SIRT1-7	Pre-clinical and clinical	Bonkowski and Sinclair, 2016; Zhao et al., 2021a
Nicotinamide riboside	NAD+ precursor Activates SIRT1-7	Pre-clinical and clinical	Fang et al., 2016
SRT1720	Synthetic Activates SIRT1	Clinical	Ren et al., 2017
SRT2104	Synthetic Activates SIRT1	Clinical	Mercken et al., 2014
Resveratrol	Natural extracts Activates SIRT1 and SIRT3	Pre-clinical and clinical	Price et al., 2012; Das et al., 2014
Irisin	Protein Activates SIRT3	Pre-clinical	Wang et al., 2020a
Quercetin	Natural extracts Activates SIRT1	Clinical	Liu et al., 2020b; Chang et al., 2021b
17b-E2	Increased SIRT1 expression	Pre-clinical	Mei et al., 2020
UBCS039	Activates SIRT6	Pre-clinical	You et al., 2017
Sirtuin inhibitors			
Nicotinamide	Inhibits SIRT1	Pre-clinical and clinical	Bonkowski and Sinclair, 2016
Melatonin	Reduced SIRT1 expression	Pre-clinical and clinical	Yi et al., 2020
Ex-527	Inhibits SIRT1	Pre-clinical	Gertz et al., 2013
AK-1	Inhibits SIRT2	Pre-clinical	Cheon et al., 2015
AK-7	Inhibits SIRT2	Pre-clinical	Chen et al., 2015
AGK2	Inhibits SIRT2	Pre-clinical	Outeiro et al., 2007

However, there are still many unresolved issues. First, while there is substantial evidence implicating sirtuins in delayed aging and suppression of the aging phenotype through activation of mitophagy, there are few experiments directly demonstrating this pathway. Secondly, the effects of different sirtuin family members on mitophagy and the mechanisms of sirtuin-induced mitophagy in aging remain poorly understood. Sirtuin family members are redundant in regulating lifespan and whether other enzyme activities (excluding acetylation activity) are involved in the aging process. Thirdly, sirtuin in different tissues seems to have different effects. The specificity of sirtuin-induced mitophagy for different aging tissues and age-related diseases also merits further investigation. Fourthly, cancer cells often use mitophagy to maintain their metabolic reprogramming and growth. This is a negative effect of sirtuin-mediated mitophagy. This raises the question that whether activation of mitophagy promotes the growth of cancer cells. Fifthly, it is still unclear about the pharmacokinetics and pharmacodynamics of SIRT activator NAD^+^ precursors and the mechanism of their transport through cell membranes into the blood and cells. Hopefully, these questions will be addressed in the future and provide a clearer direction for delaying human aging.

## Author Contributions

WW and FH contributed to the conception of the study. Related articles were screened and analyzed by WW. The manuscript of this review was prepared by WW. FH, PF, CL, FD, and SC critically revised the draft before submission. JY and XW helped perform the analysis with constructive discussions. All authors contributed to the article and approved the submitted version.

## Funding

This work was supported by grants from the National Natural Science Foundation of China (81760261, 82060219, and 81860259), Natural Science Foundation of Jiangxi Province (20202BAB206033 and 20202BABL206016), and Youth Team Project of the Second Affiliated Hospital of Nanchang University (2019YNTD12003).

## Conflict of Interest

The authors declare that the research was conducted in the absence of any commercial or financial relationships that could be construed as a potential conflict of interest.

## Publisher's Note

All claims expressed in this article are solely those of the authors and do not necessarily represent those of their affiliated organizations, or those of the publisher, the editors and the reviewers. Any product that may be evaluated in this article, or claim that may be made by its manufacturer, is not guaranteed or endorsed by the publisher.
